# Priming Is Dispensable for NLRP3 Inflammasome Activation in Human Monocytes *In Vitro*

**DOI:** 10.3389/fimmu.2020.565924

**Published:** 2020-09-30

**Authors:** Anna Gritsenko, Shi Yu, Fatima Martin-Sanchez, Ines Diaz-del-Olmo, Eva-Maria Nichols, Daniel M. Davis, David Brough, Gloria Lopez-Castejon

**Affiliations:** ^1^Division of Infection, Immunity and Respiratory Medicine, Faculty of Biology, Medicine and Health, Lydia Becker Institute of Immunology and Inflammation, University of Manchester, Manchester Academic Health Science Centre, Manchester, United Kingdom; ^2^Division of Neuroscience and Experimental Psychology, Faculty of Biology, Medicine and Health, Lydia Becker Institute of Immunology and Inflammation, University of Manchester, Manchester Academic Health Science Centre, Manchester, United Kingdom; ^3^Adaptive Immunity Research Unit, GSK, Stevenage, United Kingdom

**Keywords:** macrophage, inflammasome, NLRP3, priming, monocytes, IL-18, GSDMD

## Abstract

Interleukin (IL)-18 and IL-1β are potent pro-inflammatory cytokines that contribute to inflammatory conditions such as rheumatoid arthritis and Alzheimer’s disease. They are produced as inactive precursors that are activated by large macromolecular complexes called inflammasomes upon sensing damage or pathogenic signals. NLRP3 inflammasome activation is regarded to require a priming step that causes NLRP3 and IL-1β gene upregulation, and also NLRP3 post-translational licencing. A subsequent activation step leads to the assembly of the complex and the cleavage of pro-IL-18 and pro-IL-1β by caspase-1 into their mature forms, allowing their release. Here we show that human monocytes, but not monocyte derived macrophages, are able to form canonical NLRP3 inflammasomes in the absence of priming. NLRP3 activator nigericin caused the processing and release of constitutively expressed IL-18 in an unprimed setting. This was mediated by the canonical NLRP3 inflammasome that was dependent on K^+^ and Cl^−^ efflux and led to ASC oligomerization, caspase-1 and Gasdermin-D (GSDMD) cleavage. IL-18 release was impaired by the NLRP3 inhibitor MCC950 and by the absence of NLRP3, but also by deficiency of GSDMD, suggesting that pyroptosis is the mechanism of release. This work highlights the readiness of the NLRP3 inflammasome to assemble in the absence of priming in human monocytes and hence contribute to the very early stages of the inflammatory response when IL-1β has not yet been produced. It is important to consider the unprimed setting when researching the mechanisms of NLRP3 activation, as to not overshadow the pathways that occur in the absence of priming stimuli, which might only enhance this response.

## Introduction

Inflammasomes are molecular complexes formed by immune cells such as macrophages and monocytes in response to tissue injury or infection (1). Inflammasomes are required to process proinflammatory cytokine precursors of the Interleukin (IL-1) family, such as pro-IL-1β and pro-IL-18, into their mature and secreted active forms (mIL-1β and mIL-18), thus initiating inflammation (2). NOD-like receptor pyrin domain-containing protein 3 (NLRP3) is the best studied inflammasome sensor, the activation of which can occur in sterile inflammation. Its dysregulation is suggested to play a role in the progression of non-communicable diseases such as rheumatoid arthritis, Alzheimer’s disease, and cancer (3).

Classical or canonical NLRP3 inflammasome activation is considered to be a two-step process. The first or priming step is achieved through the activation of the nuclear factor kappa B (NF-κB) pathway, leading to the upregulation of NLRP3 and pro-IL-1β proteins (4) and to changes in NLRP3 post-translational modifications (PTMs) such as ubiquitination (5) and phosphorylation (6) that licence NLRP3 and promote inflammasome assembly. This step can be initiated by pathogen-associated molecular patterns (PAMPs) or damage-associated molecular patterns (DAMPs) binding toll like receptors (TLRs), or IL-1β and TNF-α binding to their respective receptors (7). The second or activating step leads to changes in NLRP3 conformation (8) and PTMs (9, 10) that allow NLRP3 oligomerization and consequently inflammasome activation. This step can be induced by a broad range of factors including PAMPs and DAMPs, e.g. nigericin toxin, extracellular ATP, as well as lysosomal destabilization agents such as silica and cholesterol crystals (1). Upon activation, oligomerized NLRP3 polymerizes the adaptor protein ASC, recruiting pro-caspase-1, which undergoes proximity-dependent auto-activation and as a result cleaves pro-IL-18 and pro-IL-1β into their mature forms. Simultaneously, mature caspase-1 induces the cleavage of gasdermin-D (GSDMD) (11) into N-terminal fragments that form lytic pores, facilitating the release of mature IL-1β and IL-18 as well as promoting pyroptotic cell death (12). GSDMD dependent lytic cell death also leads to the release of damage associated molecules such as high-mobility group box 1 (HMGB1), ATP, DNA and even inflammasome components themselves, e.g. ASC, which are able to propagate inflammation (13).

Although the current dogma is that the formation of an active NLRP3 inflammasome is a two-step process (14), this is not always the case. Human monocytes, in response to just the priming signal LPS, can induce the release of IL-1β in a NLRP3 dependent manner in a process that has been described as the alternative NLRP3 inflammasome (15). While pro-IL-1β is not expressed basally, pro-IL-18 is constitutively expressed in different cell types including macrophages, monocytes, dendritic cells, astrocytes and microglia (16). There are several reports that have described caspase-1 activation and IL-18 release in the absence of a priming step and just in response to the second or activating signal, especially in human cells (17–21). Despite this evidence, whether this process is dependent on the NLRP3 inflammasome has not been explored. Here, we report that primary human monocytes are equipped with a ready to assemble NLRP3 inflammasome that leads to the processing and release of constitutively expressed caspase-1 substrates such as IL-18 and GSDMD in response to just an activating signal in a sterile setting. This pathway is dependent on NLRP3, ion efflux and PTMs, which are hallmarks of the classical primed inflammasome activation. These results highlight the fact that priming might not be as necessary as initially thought for the assembly of an active inflammasome although it is required to potentiate the inflammasome response and generate IL-1β dependent inflammatory pathways.

## Experimental Procedures 

LPS (*Escherichia coli* 026:B6); nigericin sodium salt (N7143); protease inhibitor cocktail (P8340); penicillin-streptomycin (Pen/Strep, P4333); MCC950 (PZ0280); Punicalagin (P0023), 5Z-7-Oxozeaenol (O9890), Ca-074-Me (C5857), SB220025 (S9070), and Z-VAD-FMK (V116-2MG) were obtained from Sigma. JSH-23 (CAY15036) was sourced from Cambridge Bioscience. Fetal bovine serum (FBS) was purchased from Gibco. 5-Nitro-2-(3-phenylpropylamino)benzoic acid (NPPB, 0593) was sourced from Calbiochem.

Primary antibodies for Western blot assays and their final concentrations were as follows: anti‐human IL-18 (0.5 μg/ml, rabbit polyclonal, LifeSpan BioSciences, LS‐C313397), anti-human IL‐1β (0.1 μg/ml, goat polyclonal, R&D Systems, AF‐201‐NA), anti‐human caspase‐1 p20 (1:1000, mouse monoclonal, Cell Signalling Technology, 3866), anti‐NLRP3 (1 μg/ml, mouse monoclonal, Adipogen, AG‐20B‐0014), anti-NLRP3 (1:1000, Sigma, HPA012878), anti‐β‐actin‐HRP (0.2 μg/ml, mouse monoclonal, Sigma, A3854), anti-human UBE2L3 (0.2 μg/ml, mouse monoclonal, Santa Cruz, sc-390032), anti-human GSDMD (0.07 μg/ml, Novus Biologicals, NBP2-33422), anti-human IκBα (1:1000, Cell Signalling Technology, 4812). HRP conjugated secondary antibodies used for Western blotting were anti‐rabbit‐HRP (0.25 μg/ml, goat polyclonal, Dako, P0448), anti‐mouse‐HRP (1.3 μg/ml, rabbit polyclonal, Dako, P0260), and anti‐goat‐HRP (0.13 μg/ml, rabbit polyclonal, Sigma, A5420). We used the Adipogen (AG‐20B‐001) anti-NLRP3 antibody to detect NLRP3 in THP-1 cells and the anti-NLRP3 from Sigma (HPA012878) to detect NLRP3 from human blood monocytes by western blot.

### Cell Culture and Treatments

THP‐1 cells were cultured in complete media (RPMI-1640 supplemented with 2 mM L-glutamine, 10% FBS and Pen/Strep (100 U/ml)) and plated at a density of 1 × 10^6^ cells/ml. Leukocyte cones were obtained from the National Blood Transfusion Service (Manchester, UK) with full ethical approval from the Research Governance, Ethics, and Integrity Committee at the University of Manchester (ref. 2018-2696-5711). Fresh blood was isolated from healthy volunteers following approval from Ethics Committee 05/Q0401/108 and 2017-2551-3945 (University of Manchester). In both cases, PBMCs were isolated from blood by density centrifugation using a 30% Ficoll gradient. The PBMC layer was separated and washed with MACS buffer (PBS, 0.5% BSA, 2 mM EDTA) to remove platelets. Monocytes were positively selected from PBMCs from leukocyte cones with magnetic CD14^+^ MicroBeads (Miltenyi, 130‐050‐201) for 15 min at 4°C and eluted using a LS column (Miltenyi, 130‐042‐401). To differentiate monocyte‐derived macrophages (MDMs), monocytes were plated for 7 days (at a concentration of 5 × 10^5^ cells/ml) in RPMI-1640 supplemented with 2 mM L-glutamine, 10% FBS, Pen/Strep (100 U/ml) and 0.5 ng/ml M‐CSF (Peprotech, 300‐25). On day 3, half of the media was removed and replaced with fresh media.

GSDMD knockout THP-1 cells were lentivirally generated using guide RNA oligonucleotide sequences 5’-CACCGACCAGCCTGCAGAGCTCCAC-3’ and 5’-AAACGTGGAGCTCTGCAGGCTGGTC-3’ (22) by utilizing the lentiCRISPR v2 plasmid system. lentiCRISPR v2 was a gift from Feng Zhang (Addgene plasmid #52961; http://n2t.net/addgene:52961; RRID : Addgene_52961). NLRP3 deficient THP-1 cells were a gift from Prof Veit Hornung (Ludwig Maximilian University of Munich).

When comparing primed and unprimed inflammasome responses, cells were seeded in the presence/absence of LPS (1 μg/ml) for 4 h in complete media. The priming stimulus was then removed and replaced with ET buffer (147 mM NaCl, 10 mM HEPES, 13 mM d-glucose, 2 mM KCl, 2 mM CaCl_2_, and 1 mM MgCl_2_). When priming experiments were not performed in parallel, cells were plated directly into ET buffer. Cells were then treated with nigericin toxin (10 μM, 45 min or 2 h as indicated) to activate the NLRP3 inflammasome.

### Cell Death Assay

Cell death was measured using quantitative assay for the release of lactate dehydrogenase (LDH) into cell supernatants. The supernatant was gently centrifuged for 5 min at 500*g* at 4°C to remove any remaining cells. LDH release in cell supernatants were measured using CytoTox 96^®^ Non-Radioactive Cytotoxicity Assay (G1780, Promega), according to the manufacturer’s instructions. Absorbance values were recorded at 490 nm and the results were expressed as a percentage of LDH release normalized to total lysis.

### Enzyme‐Linked Immunosorbent Assay (ELISA)

Levels of human IL‐1β (DY201) and IL‐18 (DY318) were measured in the cell supernatants using ELISA kits from R&D Systems. Human IL-6 (# 88-7066-86) and TNF-α (# 88-7346-86) were detected using Invitrogen ELISA kits. ELISAs were performed following the manufacturer’s instructions.

### Western Blot

Cells were lysed on ice using a RIPA lysis buffer (50 mM Tris–HCl, pH 8, 150 mM NaCl, 1% NP‐40, 0.5% sodium deoxycholate, and 0.1% sodium dodecyl sulphate [SDS]), supplemented with a protease inhibitors cocktail (Sigma-Aldrich, P8340, 1:100). Lysates were then clarified at 21,000*g* for 10 min in order to remove the insoluble fraction. Protein concentrations of each sample were measured using BCA assays (Thermo Scientific Pierce, 23225), following the manufacturer’s guidelines, so an equal amount of protein was loaded for each sample. Cell supernatants were centrifuged at 500*g* for 5 min to remove dead cells and concentrated using 10 kDa MW cut‐off filters (Amicon, Merck Millipore), as described by the manufacturer. Supernatants and whole‐cell lysates were diluted in 1× reducing Laemmli buffer containing 1% β‐mercaptoethanol. Samples were boiled at 95°C for 5 min and separated by Tris-glycine SDS-PAGE. Proteins were transferred onto nitrocellulose membranes (0.2 µm) and blocked in PBS‐Tween (PBS-T, 0.1%) containing 5% skimmed milk for 1 h at room temperature. Membranes were then incubated overnight with the specific primary antibody in blocking buffer at 4°C. following day, membranes were labeled with a horseradish peroxidase‐conjugated secondary antibody for 1 h at room temperature. Membranes were then washed, developed and captured digitally using Clarity™ Western ECL Blotting Substrate (Bio‐Rad, 1705061) in a ChemiDoc™ MP Imager (Bio‐Rad).

### ASC Oligomerization Assay

After inflammasome stimulation, undifferentiated WT and NLRP3 KO THP-1 cells and monocytes were placed on ice. 1% (v/v) NP-40 and protease inhibitor cocktail were added directly to wells. Cell total lysates were separated into NP-40 soluble and insoluble fractions using differential centrifugation at 6,800*g* for 20 min at 4°C. The soluble fraction containing cell supernatant and lysates was utilized for western blotting analysis, whereas the NP-40 insoluble pellets were chemically crosslinked with 2 mM disuccinimidyl suberate (DSS) (Thermo Fisher) for 30 min at RT. Crosslinked pellets were further centrifuged at 6,800*g* for 20 min and resuspended in boiled 1× Laemmli buffer for standard SDS-PAGE.

### Caspase-Glo^®^ 1 Inflammasome Assay

Caspase-1 activity was measured in the supernatants using Caspase-Glo^®^ 1 Inflammasome Assay (Promega). Briefly, cell supernatants were combined with Z-WEHD aminoluciferin substrate and illuminescence measured following 1 h incubation.

### Cell Vitality Assay

Cells were stimulated as required, washed in 1× PBS, and resuspended at 1 × 10^6^ cells/ml in PBS. Cells were then stained with LIVE/DEAD Cell Vitality Assay Kit (L34951) with C12-Resazurin and SYTOX ^®^ Green Stain (Thermo Scientific) as per manufacturer’s instructions. 100,000 cells per sample were acquired using a 3-laser Fortessa with BD FACSDiva software and analyzed with FlowJo software (version 10, TreeStar).

### Statistical Analysis

GraphPad Prism 8 software was used to carry out all statistical analysis. Differences between 2 groups were analyzed using t-test. Differences between 3+ groups were analyzed using one‐way ANOVA with the *post hoc* Dunnett’s test or two‐way ANOVA with the *post hoc* Tukey’s test for multiple comparisons. Data was shown as mean +/- standard deviation (S.D.). Accepted levels of significance were *P < 0.05, **P < 0.01, ***P < 0.001, ****P < 0.0001.

## Results

### Priming Is Not Required for NLRP3 Inflammasome Activation in Human Monocytes *In Vitro*

**Figure 1:**
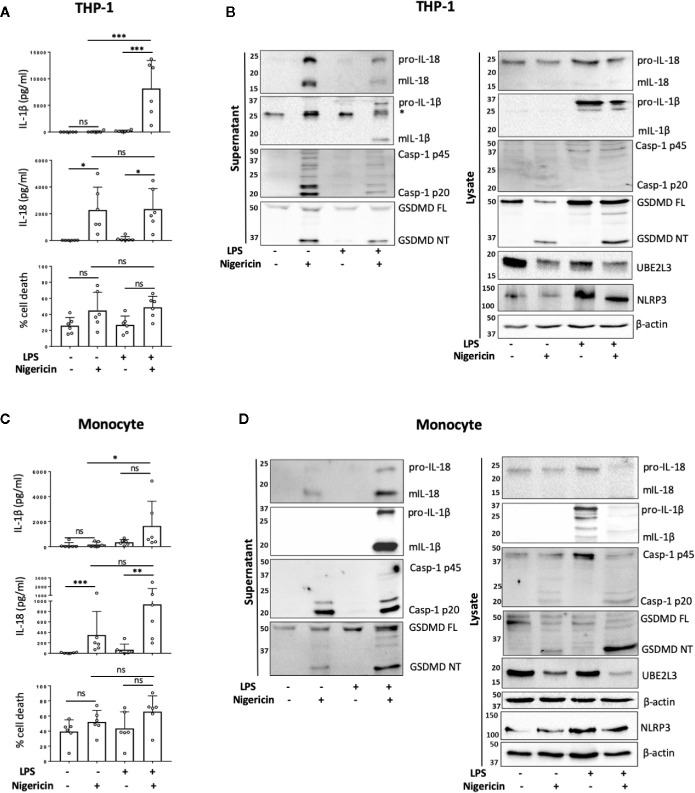
Priming is not required for NLRP3 inflammasome activation in human monocytes *in vitro*. **(A, B)** Undifferentiated THP-1 cells (n=6 independent biological replicates) and **(C, D)** primary CD14^+^ monocytes (n=6 independent biological replicates (each point represents a different blood donor)) were left untreated or primed with LPS (1 µg/ml, 4 h) prior to treatment with nigericin (10 µM, 45 min) to activate the NLRP3 inflammasome. **(A, C)** IL-1β and IL-18 were measured by ELISA and cell death was measured by LDH assay and shown as percentage relative to total cell death, mean ± S.D., *P < 0.05; **P < 0.01; ***P < 0.001; ns (non significant) using one-way ANOVA comparing all groups. **(B, D)** Western blot analysis for mIL-18 (18 kDa), pro-IL-18 (24 kDa), mIL-1β (17 kDa), pro-IL-1β (34 kDa), mCaspase-1 (20 kDa), pro-Caspase-1 (45 kDa), GSDMD full length (FL, 53 kDa), GSDMD N-terminus (NT, 31 kDa), UBE2L3 (17.9 kDa), NLRP3 (113 kDa), as well as loading control β-actin (42 kDa). Blots are representative of at least 3 independent biological experiments and in case of monocytes 3 different blood donors.

To determine if NLRP3 inflammasome activation occurs in human monocytes in the absence of priming, we first compared the release of mIL-18 and mIL-1β, as pro-IL-18 gene expression is constitutive, while pro-IL-1β requires upregulation by priming (23). Undifferentiated THP-1 cells were primed with LPS (1 µg/ml) for 4 h, or left unprimed, followed by treatment with the NLRP3 activator nigericin (10 µM, 45 min). Nigericin induced mIL-18 release and cell death in both primed and unprimed cells to the same extent, while it only induced mIL-1β release in LPS primed THP-1 cells (**Figures 1A, B**). In line with this, caspase-1 cleavage into its active form (p20) was also detected in both primed and unprimed cells, as well as IL-18 processing and release (**Figure 1B**). Nigericin treatment, with or without priming, led to GSDMD cleavage, detected by the appearance of a GSDMD fragment of 31 kDa, corresponding to the pore-forming N-terminus (GSDMD NT) (**Figure 1B**). GSDMD cleavage is a consequence of caspase-1 activation and is required for pyroptosis (11). However, we could not detect statistically significant elevated levels of cell death following nigericin treatment by measuring LDH release (**Figure 1A**) or by uptake of the SYTOX Green nuclear dye (**Figure S1**). Caspase-1 activation leads to the direct or indirect processing of substrates other than IL-1β, IL-18, and GSDMD (24–26). For example, caspase-1 can mediate the degradation of the E2 ubiquitin-conjugating enzyme UBE2L3 involved in NF-κB activation and pro-IL-1β turnover (27). To assess if active caspase-1 in unprimed cells can be involved in other cellular processes, we investigated whether the second signal alone was sufficient to induce degradation of UBE2L3. Nigericin treatment alone was enough to reduce UBE2L3 levels, suggesting that caspase-1 is active and able to cleave substrates other than IL-1β and IL-18 (**Figure 1B**). The NLRP3 protein was detected in THP-1 cells without any treatment, and was upregulated following exposure to LPS (**Figure 1B**). Despite this, caspase-1 and GSDMD cleavage, UBE2L3 degradation and mIL-18 release were not potentiated by LPS stimulation in THP-1 cells suggesting that changes in NLRP3 expression are not solely responsible for intensity of inflammasome activation. Overall, this highlights that inflammasome activation without prior priming can lead to the cleavage of a number of constitutively expressed caspase-1 substrates.

Next we tested whether unprimed inflammasome activation occurred in freshly isolated primary human CD14^+^ monocytes from healthy donors. We observed that the treatment of CD14^+^ human monocytes with nigericin alone was sufficient to trigger significant mIL-18 secretion (**Figures 1C, D**). Western blot analysis revealed that, like THP-1 cells, unprimed monocytes responded to nigericin treatment with caspase-1 activation, GSDMD cleavage, UBE2L3 degradation and the release of IL-18, but not IL-1β (**Figures 1C, D**). We observed that priming human monocytes with LPS, unlike THP-1 cells, potentiated nigericin-induced secretion of mIL-18 and cell death, although this was not significantly different to the unprimed response (**Figure 1C**). We also observed a slight increase in NLRP3 levels after LPS priming, as was also the case in THP-1 cells (**Figure 1D**).

To exclude the possibility of a priming effect by engagement of CD14 receptor during monocyte purification, we also analyzed IL-18 release and cell death in response to nigericin in the CD14 negative peripheral blood mononuclear cell (PBMC) fraction in the absence of LPS priming. Similarly to CD14^+^ monocytes, we also found mIL-18 release in response to nigericin, although at much lower levels likely due to the lower number of inflammasome forming cells in this fraction (**Figure S2A**). As most experiments were carried out on cells isolated from leukocyte cones, we wanted to ensure that packaging and storing blood in these cones did not prime the cells. We therefore tested freshly isolated PBMCs from healthy volunteers. When stimulated with nigericin, these PBMCs showed unprimed IL-18 release (**Figure S2B**). This suggests that inflammasome activation occurs in recently isolated, not previously packaged cells that also have not undergone CD14^+^ magnetic separation. Small levels of IL-1β release were detected in unprimed PBMCs, which could be explained by higher pro-IL-1β expression in human PBMCs compared to monocytes and macrophages (28).

To determine whether unprimed NLRP3 inflammasome assembly is unique to monocytes, we also carried out parallel experiments in human monocyte derived macrophages (MDMs). We observed non-significant mIL-18 release from MDMs in the absence of priming (**Figure S3A**). The levels of secreted mIL-18 released by LPS primed cells activated with nigericin were significantly higher than in unprimed MDMs. Unlike in primary monocytes, the cleavage of caspase-1 by inflammasome activation without LPS stimulation was also much lower than in LPS primed cells (**Figures S3B, C**). The cleavage of GSDMD and maturation of IL-18 occurred without priming at lower levels than in LPS primed cells (**Figure S3B**). UBE2L3 did not appear to be degraded without LPS priming. Therefore, although there is a small response to inflammasome activation in human macrophages, unlike monocytes, priming is required for a full response.

### TAK1 and NF-κB Differentially Contribute to Unprimed Inflammasome Activation

**Figure 2:**
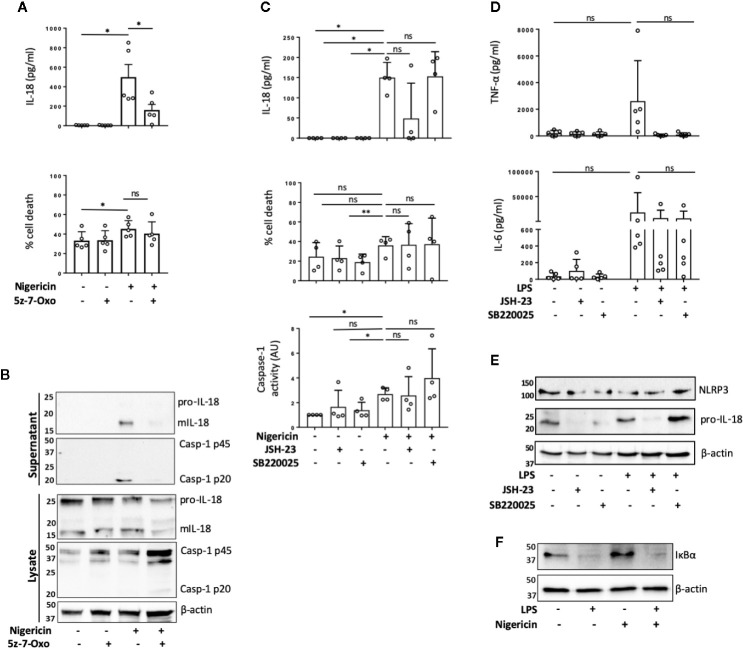
TAK1 and NF-κB differentially contribute to unprimed inflammasome activation in human monocytes. **(A, B)** Primary human monocytes were pre-incubated with 5z-7-Oxozeaenol (0.5 µM) for 15 min prior to treatment with nigericin (10 µM, 45 min). **(A)** Secreted IL-18 was measured by ELISA and cell death was measured by LDH assay and shown as percentage relative to total cell death. n=5 independent biological replicates (each point represents a different blood donor). Error bars represent mean ± S.D., *P < 0.05 using one-way ANOVA comparing each sample to the nigericin only treated sample. **(B)** Supernatants and lysates were analyzed for mIL-18 (18 kDa), pro-IL-18 (24 kDa), mCaspase-1 (20 kDa), pro-Caspase-1 (45 kDa), as well as loading control β-actin (42 kDa). Blots are representative of at least 3 independent experiments. **(C)** Primary human monocytes were left untreated or pre-treated with JSH-23 (40 µM, 13 h) or SB220025 (20 µM, 30 min) followed by treatment with nigericin (10 µM, 45 min). Cell supernatants were assayed for the release of IL-18, cell death by LDH release and caspase-1 activity by caspase-1-Glo assay. n=4 independent biological replicates (each point represents a different blood donor). Error bars represent mean ± S.D., * = P < 0.05; ** = P < 0.01; ns (not significant) using one-way ANOVA comparing each sample to the nigericin only treated sample. **(D, E)** Primary human monocytes were left untreated or pre-treated with JSH-23 (40 µM, 13 h) or SB220025 (20 µM, 30 min) following treatment with LPS (1 µg/ml, 4 h) or left untreated. **(D)** Cell supernatants were assayed for TNF-α and IL-6 release. n=5 independent biological replicates (each point represents a different blood donor). Error bars represent mean ± S.D., *P < 0.05 using one-way ANOVA comparing each sample to the LPS only treated sample. **(E)** Lysates were analyzed for proIL-18 (24 kDa) and NLRP3 (113 kDa) as well as loading control β-actin (42 kDa). **(F)** Western blot analysis of human monocyte lysates for IκBα (39 kDa) and loading control β-actin (42 kDa) from cells left untreated or treated with nigericin (10 µM, 45 min). All blots are representative of 2 independent biological experiments (each from a different blood donor).

TAK1 (transforming growth factor beta-activated kinase 1) is an upstream regulator of the crucial intracellular kinases: I-kappa B kinase complex (IKK), p38, and JNK that mediate activation of the transcription factors NF-κB and AP-1, thus contributing to the priming step of NLRP3 inflammasome formation (4, 29). TAK1 is also activated in response to inflammasome activating signals such as the lysosomotropic agent Leu-Leu-O-methyl ester (LLMe) (30) or hypotonic stress-induced cell volume change (31) and mediates TLR induced non-transcriptional activation of the NLRP3 inflammasome (32). However, whether TAK1 controls NLRP3 inflammasome activation in the absence of TLR4 sensitization has not been explored. To test whether TAK1 was involved in unprimed NLRP3 activation, we pre-treated human monocytes with an inhibitor of TAK1 catalytic activity: 5z-7-oxozeaenol (5z-7) (0.5 µM) for 15 min prior to nigericin stimulation. We found that TAK1 inhibition significantly impaired mIL-18 release in primary human monocytes (**Figures 2A, B**) as well as reduced the cleavage of caspase-1 into its mature form (p20) (**Figure 2B**). However, TAK1 inhibition did not alter unprimed inflammasome activation in THP-1 cells (**Figure S4A**).

To assess if TAK1 contributed to unprimed inflammasome activation through NF-κB or p38 pathways, we treated human monocytes with either the NF-κB nuclear translocation inhibitor JSH-23 (33) (40 µM, 13 h) or p38 inhibitor SB220025 (34) (20 µM, 30 min) followed by nigericin or vehicle treatment (10 µM, 45 min). We found that in human monocytes, JSH-23 treatment did not significantly affect IL-18 release, cell death or caspase-1 activity (**Figure 2C**) in response to nigericin. However, JSH-23 treatment in THP-1 cells led to a reduction in IL-18 release, cell death and caspase-1 activation (**Figure S4B**). As expected, LPS treatment (1 μg/ml, 4 h) induced the release of IL-6 and TNF-α in primary monocytes and TNF-α in THP-1 cells. Minimal levels of IL-6 and TNF-α were found in the supernatants of untreated cells, suggesting that the isolated monocytes and THP-1 cells are unprimed without LPS treatment (**Figure 2D**, **Figure S4C**). Pre-treatment with JSH-23 reduced LPS induced IL-6 and TNF-α release in both cell types although this was not statistically significant in human monocytes given the high variability among monocytes from different donors (**Figure 2D**, **Figure S4C**). When we normalized the data to the maximum IL-6 or TNF-α release induced by LPS alone in each donor, we observed that the levels of IL-6 were reduced to 67.4% ±; 21.4% (mean ±; S.D.; p=0.02) and the levels of TNF-α release reduced to 4.7% ±; 4.3% (mean ±; S.D.; p=0.054) (Friedman test, *post hoc* Dunn’s test), indicating a clear effect of JSH-23 in these cells. JSH-23 did not affect NLRP3 levels in either human monocytes nor THP-1 cells. However, while JSH-23 reduced pro-IL-18 levels in human monocytes (**Figure 2E**), it did not alter proIL-18 levels in THP-1 cells (**Figure S2D**). This could explain why we observed reduced levels of nigericin-induced IL-18 release in several donors compared to untreated cells. To exclude the possibility that nigericin itself was activating NF-κB, we assessed the degradation of IκBα in the presence or absence of nigericin or LPS. While LPS treatment reduced IκBα levels, nigericin stimulation resulted in no changes in IκBα levels in either human monocytes or THP-1 cells (**Figure 2F**, **Figure S4E**). Inhibition of p38 with SB220025 did not affect IL-18 release in response to nigericin in human monocytes or THP-1 cells despite impairing IL-6 and TNF-α release induced by LPS (1 μg/ml, 4 h) treatment (**Figures 2C, D**; **Figures S4B, C**). In this case, when we normalized the data to the maximum IL-6 or TNF-α release level induced by LPS alone in each donor, we observed that SB220025 reduced IL-6 levels to 65.2% ±; 21.4% (mean ±; S.D.; p=0.054) and TNF-α release to 3% ±; 2.5% (mean ±; S.D.; p=0.02) (Friedman test, *post hoc* Dunn’s test), validating that the inhibitor works on human monocytes. These results suggests that TAK1 and NF-κB might be active at basal levels in human monocytes and THP-1s and differentially contribute to the unprimed inflammasome response, while MAPK p38 activity may not be necessary.

### mIL-18 Release in the Absence of Priming Is NLRP3-Dependent

**Figure 3:**
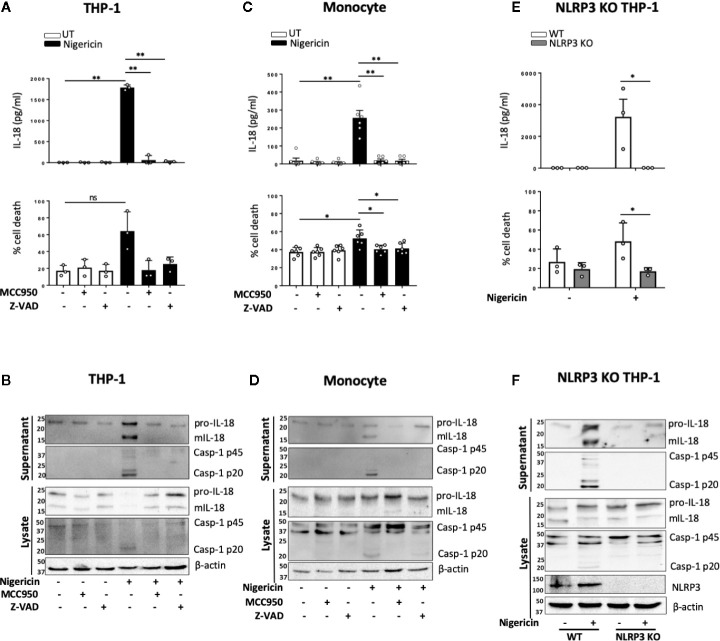
mIL-18 release in the absence of priming is NLRP3 dependent. THP-1 cells **(A, B)** and monocytes **(C, D)** were pre-incubated with MCC950 (1 µM) or Z-VAD (50 µM) for 15 min prior to treatment with nigericin (10 µM, 45 min). **(A, C)** Secreted IL-18 was measured by ELISA and cell death was measured by LDH assay and shown as percentage relative to total cell death, n=3 independent biological replicates for THP-1 and n=6 independent blood donors for human monocytes, mean ± S.D., *P < 0.05; **P < 0.01; ns (not significant) using one-way ANOVA comparing each sample to the nigericin only treated sample. **(E, F)** Unprimed WT or NLRP3 KO THP-1 were treated with nigericin (10 µM, 45 min). **(E)** Secreted IL-18 was measured by ELISA and cell death was measured by LDH assay and shown as percentage relative to total cell death, n=3 independent biological replicates, mean ± S.D., * = P < 0.05; ns (not significant) using two-way ANOVA comparing nigericin treated WT and NLRP3 KO THP-1s. **(B, D, F)** Western blot analysis for mIL-18 (18 kDa), pro-IL-18 (24 kDa), mCaspase-1 (20 kDa), pro-Caspase-1 (45 kDa), and in some cases NLRP3 (113 kDa), as well as loading control β-actin (42 kDa). Blots are representative of at least 3 independent biological experiments and blood donors.

Unprimed release of mIL-18 in response to nigericin has initially been described, prior to discovery of the NLRP3 inflammasome, as a necrosis dependent pathway mediated by cathepsin B and lysosomal disruption but independent of caspase-1 activity (17). As cathepsin B and lysosomal destabilization have been closely linked to inflammasome activation, we first tested the effect of cathepsin B inhibitor Ca-074-Me on unprimed IL-18 release in THP-1 cells. However, we did not observe any inhibition of nigericin induced mIL-18 release in the presence of cathepsin B inhibitor (**Figure S5**). Upon treating THP-1 cells (**Figures 3A, B**) or primary monocytes (**Figures 3C, D**) with NLRP3 inhibitor MCC950 (1 μM, 15 min) prior to the addition of nigericin (10 μM, 45 min), we found that MCC950 inhibited nigericin-induced mIL-18 release, caspase-1 cleavage into its active form, and cell death in both cell types. Similar effects were observed when either human monocytes or THP-1 cells were pre-treated with an irreversible pan-caspase inhibitor Z-VAD-FMK (ZVAD, 50 μM, 15 min) (**Figures 3A–D**). To further confirm the involvement of NLRP3 in this process, we utilized NLRP3 knock out (KO) THP-1 cells and revealed that IL-18 processing and release, caspase-1 activation and cell death following unprimed inflammasome activation did not occur in NLRP3 deficient cells compared to WT (**Figures 3E, F**).

### Unprimed NLRP3 Inflammasome Activation Has the Same Requirements as the Canonical Inflammasome

**Figure 4:**
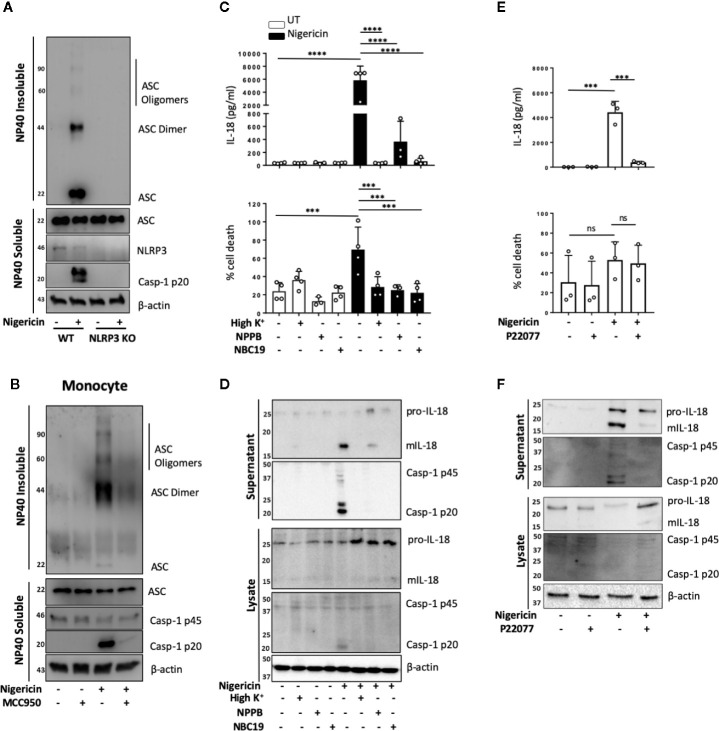
Unprimed NLRP3 inflammasome activation has the same requirements as the canonical inflammasome. **(A)** WT and NLRP3 KO THP-1 cells were left unprimed prior to treatment with nigericin (10 µM, 2 h) to activate NLRP3 inflammasome. **(B)** Primary human monocytes were pre-incubated with MCC950 (1 µM) for 15 min prior to treatment with nigericin (10 µM, 45 min). The NP-40 soluble fraction and DSS crosslinked insoluble fraction were immunoblotted for ASC monomers, dimers and oligomers. Blots are representative of 3 independent biological experiments and blood donors. **(C, D)** THP-1 cells were left unprimed and pre-incubated with high K^+^ buffer, NPPB (50 µM) or NBC19 (25 µM) for 15 min prior to treatment with nigericin (10 µM, 45 min). **(E, F)** THP-1 cells were left unprimed pre-incubated with P22077 (2.5 µM, 15 min) prior to treatment with nigericin (10 µM, 45 min). n=3 independent biological replicates, mean ±; S.D., ***P < 0.001; ****P < 0.0001; ns (not significant) using one-way ANOVA comparing each sample to nigericin only treated sample. **(D, F)** Supernatants and lysates were analyzed for mIL-18 (18 kDa), pro-IL-18 (24 kDa), mCaspase-1 (20 kDa), pro-Caspase-1 (45 kDa), as well as loading control β-actin (42 kDa). Blots are representative of at least 3 independent experiments and in the case of primary monocytes, blood donors.

NLRP3 inflammasome activation in monocytes can occur through different pathways (35). In primed monocytes, nigericin is a trigger of canonical NLRP3 inflammasome activation, which is characterized by the oligomerization of ASC (36). We therefore tested whether ASC oligomerization also occurred in unprimed monocytes. We found that nigericin treatment (10 μM, 2 h) of unprimed THP-1 cells resulted in ASC oligomerization that was detected in the crosslinked NP-40 insoluble fraction of the total lysate, and that this was absent in NLRP3 KO cells (**Figure 4A**). Similarly, unprimed primary human monocytes formed ASC oligomers following stimulation with nigericin and this did not occur in monocytes pre-treated with the NLRP3 inhibitor MCC950 (10 μM, 15 min) (**Figure 4B**).

As well as ASC oligomerization, the classical primed canonical NLRP3 inflammasome activation in response to nigericin is dependent on potassium (K^+^) (37) and chloride (Cl^−^) (38) efflux. This is unlike the alternative NLRP3 inflammasome that occurs in human monocytes and assembles following prolonged priming signals in a K^+^ (15) and Cl^−^ independent manner (38). We tested the requirement of K^+^ and Cl^−^ efflux during unprimed inflammasome activation in response to nigericin. We incubated unprimed THP-1 cells either in high K^+^ containing media or pre-treated these cells with broad-spectrum chloride channel inhibitor NPPB (50 μM), or NBC19: a recently described NLRP3 inhibitor (25 μM) (39), as a positive control, for 15 min prior to nigericin treatment (**Figures 4C, D**). This revealed that unprimed mIL-18 release, caspase-1 cleavage and cell death following nigericin stimulation were dependent on K^+^ efflux and were also sensitive to Cl^−^ channel inhibitor NPPB (**Figures 4C, D**), as they were reduced to the same extent as in the presence of inflammasome inhibitor NBC19.

Deubiquitination of NLRP3 by the priming and activating step has been described as necessary for the formation of an active NLRP3 inflammasome (5). We have recently shown that deubiquitinases USP7 and USP47 mediate canonical NLRP3 inflammasome activation in human macrophages independently of transcriptional priming (9). Hence we hypothesized that DUBs might also mediate inflammasome activation in an unprimed context. To test this, we investigated the effect of the dual USP7/USP47 inhibitor P22077 (9) on inflammasome activation without priming. We found that pre-incubation of THP-1 cells with P22077 (2.5 µM, 15 min) prior to nigericin treatment significantly reduced IL-18 release (**Figure 4E**). This was accompanied by a reduction in caspase-1 maturation (**Figure 4F**), although no significant reduction in cell death was observed. Similar results were found in primary human monocytes (**Figure S6**). These data show that USP7 and USP47 can control inflammasome activation independently of the priming step.

### mIL-18 Release From Unprimed Monocytes Is Dependent on GSDMD

**Figure 5:**
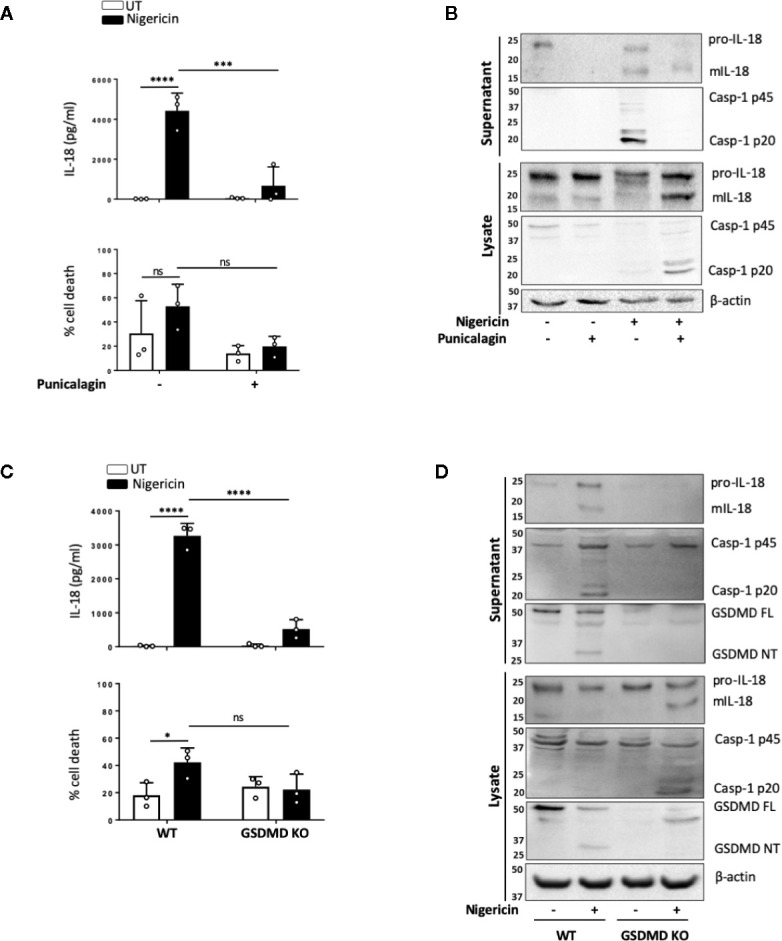
mIL-18 release from unprimed monocytes is dependent on GSDMD. **(A, B)** Unprimed THP-1 cells pre-incubated with punicalagin (50 µM, 15 min) prior to treatment with nigericin (10 µM, 45 min). **(C, D)** Unprimed WT and GSDMD KO THP-1s were treated with nigericin (10 µM, 45 min). **(A, C)** Secreted IL-18 was measured by ELISA and cell death was measured by LDH assay and shown as percentage relative to total cell death, n=3 independent biological replicates, mean ± S.D., *P < 0.05; ***P <0.001; ****P <0.0001; ns (not significant) using one-way ANOVA comparing each sample to nigericin only treated sample **(A)** or two-way ANOVA comparing UT to nigericin treated WT THP-1s as well as nigericin treated WT THP-1s to nigericin treated GSDMD KO THP-1s. **(C)**. **(B, D)** Western blot analysis of THP-1 cells for mIL-18 (18 kDa), pro-IL-18 (24 kDa), mCaspase-1 (20 kDa), pro-Caspase-1 (45 kDa), GSDMD full length (FL, 53 kDa), GSDMD N-terminus (NT, 31 kDa), as well as loading control β-actin (42 kDa). Blots are representative of at least 3 independent experiments.

NLRP3 inflammasome mediated IL-1 release, membrane permeabilization and pyroptotic cell death are closely associated, and these events are tightly linked to GSDMD cleavage and pore formation. However, certain NLRP3 activators such as changes in cell volume can induce mIL-1β and mIL-18 release in the absence of pyroptosis. As we observed that levels of cell death triggered by nigericin in unprimed cells were lower and more variable than those expected from murine cells, we set out to determine whether inflammasome-induced membrane permeabilization in unprimed monocytes was involved in the release of mIL-18. We first tested the effect of the polyphenolic compound punicalagin, known to inhibit plasma membrane permeability, as well as mIL-1β and mIL-18 release induced by inflammasome activation, while allowing caspase-1 activity (40, 41). In unprimed THP-1 cells, 15 min pre-treatment with 50 µM punicalagin prior to nigericin significantly blocked IL-18 release (**Figure 5A**). Western blot analysis revealed that punicalagin treated cells still displayed caspase-1 activation, indicating inflammasome activation, but the release of mature caspase-1 (p20) was blocked (**Figure 5B**). Similarly, pro-IL-18 was cleaved into its mature form but its release was inhibited by punicalagin.

The effect of punicalagin on inflammasome activation resembles that of GSDMD KO (40–42). Similar to pro-IL-18, GSDMD is constitutively expressed in unprimed THP-1 and human monocytes. As the GSDMD pore has been proposed as a mediator of mIL-1β release (42) and we observed GSDMD cleavage in unprimed monocytes, we investigated if GSDMD was required for mIL-18 release. IL-18 release caused by nigericin was significantly lower in cells lacking GSDMD compared to WT control (**Figure 5C**). Like with punicalagin treatment, GSDMD deficient cells where still able to activate caspase-1 and process pro-IL-18 into its mature form (**Figure 5D**) but its released was impaired. Overall, these data suggest that caspase-1 cleavage of GSDMD and subsequent pyroptosis may be a key mechanism for the release of inflammasome regulated cytokines following inflammasome assembly in the absence of priming.

## Discussion

Our work shows that human monocytes are able to assemble a functional NLRP3 inflammasome in response to an activating signal even in the absence of a priming step. The priming step has long been regarded as an essential stage for the assembly of an active NLRP3 inflammasome. Initially, it was believed that priming was only required to upregulate NLRP3 and pro-IL-1β proteins in response to the activation of NF-κB signaling (4). In recent years, however, a role for priming independent of transcriptional regulation has been uncovered and is believed to be mediated by PTMs such as changes in the ubiquitination or phosphorylation of NLRP3 (5, 43). Our data shows that an initial priming step is not required for the formation of an active NLRP3 canonical inflammasome but might be required to enhance inflammasome activation and the inflammatory response, depending on the cell type.

Evidence linking the ability of human monocytes to activate caspase-1 and induce the release of mIL-18 in the absence of priming was first described by Perregaux et al. (18) and Mehta et al. (20). The first group reported that ATP treatment of human blood in the absence of LPS priming leads to IL-18 release, while Mehta et al. showed that ATP treated human PBMCs activate caspase-1 in the absence of LPS priming but do not release IL-18 (20). It was later shown, in THP-1 cells and human monocytes, that lysosomal rupture induced by nigericin contributes to caspase-1 activation and IL-18 release in what was thought to be necrosis (17). In this report, IL-18 release was impaired with a cathepsin B inhibitor but not by caspase-1 inhibition. Here, we have further characterized the release of IL-18 in unprimed human monocytes and THP-1 cells and showed that it is dependent on the activation of the canonical NLRP3 inflammasome as it is a process blocked by preventing K^+^ efflux and by Cl^−^ channel inhibition. This results in the formation of ASC oligomers, caspase-1 activation and GDSMD cleavage and pyroptosis. Although we cannot completely rule out an unknown priming effect of monocytes *in vivo*, our data on THP-1 cells, human PBMCs, CD14^+^ monocytes and human CD14^-^ PBMC fraction, as well as previous reports using human blood, purified PBMCs (17, 18) and human derived DCs (19), all show the dispensability of priming for inflammasome activation *in vitro*. Moreover, primary human monocytes that were seeded and stimulated directly in serum free buffer behaved the same as those cultured in complete media, ruling out any priming effect of serum (**Figures 1C, D** vs **Figures 3C, D**). Together, this evidence suggests that unprimed inflammasome assembly is not an effect of sample preparation or cell culture conditions.

It has been previously evidenced that certain mouse cells are activated by signal 2 alone. For example, unprimed primary murine peritoneal macrophages from both LPS-sensitive FVBN mice and from LPS-unresponsive C3H/HeJ mice secrete significant levels of IL-18 in response to nigericin treatment, also suggesting inflammasome activation (17). Unprimed monocytes from C57BL/6 mice have been shown to respond to nigericin with elevated LDH release, suggesting possible inflammasome activation and pyroptosis but whether this leads to inflammasome activation has not been directly tested (15). On the contrary, mouse BMDMs appear to require a priming signal for NLRP3 inflammasome assembly, as demonstrated in a number of studies (6, 44). Taken together, this evidence suggests that both human and murine monocytes are capable of TLR independent NLRP3 activation, whereas human MDMs and murine BMDMs, but not murine peritoneal macrophages, do not have this ability. Whether this is a product of culturing cells *ex vivo* for prolonged periods of time or due to differences between cell types remains to be explored.

A feature of the canonical NLRP3 inflammasome is its regulation by kinases such as TAK1, JNK (c-Jun N-terminal kinase) and protein kinase D (PKD) (6, 45, 46) and phosphatases such as protein phosphatase 2A (PP2A), protein tyrosine phosphatase non-receptor type 22 (PTPN22) (47) and PTEN (48). Whether these enzymes contribute to the NLRP3 priming, licencing, or activating step is not clear. TAK1 kinase is described to regulate the canonical NLRP3 inflammasome at the priming level by either mediating transcriptional priming through its role in NF-κB signaling downstream of TLR4 (29) or *via* non transcriptional priming (32). TAK1 activation is also induced by NLRP3 activating signals such as hypotonicity, ATP or lysosomotropic agents (30, 31, 49) in macrophages that have been previously engaged with TLR activators. Lysomal destabilization has been previously linked to an unprimed inflammasome response and is an established activator of TAK1, downstream of which JNK regulates the NLRP3 inflammasome through the oligomerization of ASC (49). Whether TAK1 plays a role in inflammasome activation in a setting where TLR4 is not engaged has not been explored. We found that TAK1 inhibition significantly reduced inflammasome activation in unprimed human monocytes but not in THP-1 cells. TAK1 activation can be induced by only osmotic stress in mouse embryonic fibroblasts (MEFs) (50) and is closely linked to lysosomal destabilization. It is hence plausible that in human monocytes, nigericin and potentially other activating signals induce TAK1 activation independently of priming but dependent on lysosomal rupture and hence contribute to NLRP3 inflammasome assembly. It has recently been suggested that in human myeloid cells, NLRP3 inflammasome can form in the absence of NEK7, which was once considered an essential component of this inflammasome (51). This work proposes that NEK7 contributes to inflammasome activation as a priming factor, required at the initial stages of the response. This requirement is bypassed after prolonged priming by active TAK1 (51). However the roles of NEK7 and TAK1 in NLRP3 activation were not explored in an unprimed setting.

Constitutive NF-κB activation by tonic signals, independent of IL-1R, TNFR, or TLR priming, occurs in the bone marrow (52) and has also been found in human monocytes as well as mouse peritoneal and bone marrow derived macrophages. However, the mechanisms that control this basal activation are not clear (53). High constitutive NF-κB levels are also a feature of acute myeloid leukemia (AML) (54) and hence this might be reflected in THP-1 cells. We found that preventing NF-κB nuclear translocation with the inhibitor JSH-23 did not alter NLRP3 levels, but differentially affected IL-18 in human monocytes and THP-1 cells. In human monocytes, inhibition of NF-κB nuclear translocation led to a reduction in basal pro-IL-18 levels but had no effect on inflammasome activation (cell death and caspase-1 activity) while in THP-1 cells it blocked inflammasome activation without affecting IL-18 protein levels. It is possible that high basal activation of NF-κB contributes to higher levels of IL-18 and inflammasome activation observed in THP-1 cells. Our results however differ from previous work (32) that found that TAK1 inhibition impaired inflammasome activation but JSH-23 did not. These differences can be explained by the fact that our THP-1 cells have not been differentiated with PMA and primed with LPS as done by Gong et al. (32) and that we did a long pre-treatment with JSH-23 (13 h) while Gong *et al.* added the inhibitor at the same time as the NLRP3 activating stimuli nigericin or ATP (30 min). Our data also showed no degradation of IκBα in response to nigericin, indicating that contribution of NF-κB to unprimed inflammasome is not mediated by the activating signal as previously shown for LLME (55). Despite the regulation of p38 activity downstream of TAK1 and the contribution of p38 activity to NLRP3 inflammasome activation (56, 57), we observed no effect of p38 inhibition on unprimed NLRP3 inflammasome activation. Our data highlight an important role for TAK1 and basal NF-κB activity in unprimed inflammasome activation. However the mechanisms by which they do so remain unknown.

It is now evident that changes in PTMs such as ubiquitination or phosphorylation induced by priming or activating signals are essential for the assembly of an active NLRP3 inflammasome (5, 43). Both human and mouse NLRP3 have been described to require TLR4 priming for rapid deubiquitination to be able to respond to the subsequent activating signal (5). Juliana et al. also reported that cell lines overexpressing NLRP3 do not require TLR signaling to respond to an inflammasome activator. However, our results showed that human monocytes and THP-1 cells have basal NLRP3 protein levels, suggesting that this is not the reason why TLR4 engagement is needed for inflammasome assembly. Moreover, our THP-1 data reveals that increased NLRP3 protein expression is not associated with enhanced inflammasome activation. The same study also reported that in the absence of LPS, ATP (signal 2) can cause the deubiquitination of NLRP3 (5). Several deubiquitinases such as USP7, USP47, and BRCC3 have been reported to licence NLRP3 (9, 10). USP7 and USP47 upregulate their activity in response to inflammasome activating signals, even in the absence of priming, and their activity is required to form a canonical NLRP3 inflammasome independently of transcriptional priming (9). Our new data takes this a step further by showing that inhibition of USP7 and USP47 activity prevents the canonical NLRP3 inflammasome activation in unprimed human monocytes. This highlights the role of USP7 and USP47 in sensing danger independently of the priming state of the cell and their ability to mediate inflammasome formation, hence facilitating a fast inflammatory response. Further characterization of basal NLRP3 modifications, especially ubiquitination, as well the activity of various deubiquitinases across species and cell types would greatly expand our understanding of the mechanisms of inflammasome activation.

Pyroptotic cell death is a very well established feature of NLRP3 inflammasome activation. It is mediated by caspase-1 and GSDMD cleavage. Our results, however showed that cell death levels induced by nigericin in both primed and unprimed human cells (monocytes and macrophages) were lower than those traditionally observed in murine cells. This was not just due to a sensitivity issue of the LDH detection assay as cell death measured by nuclear dye uptake showed very similar results. This could be explained by some mouse to human differences, however this has not been addressed in this study and will require further investigation.

Caspase-1 has a broad range of substrate specificity and its activation by the inflammasome regulate cellular responses beyond those attributed to IL-1β and IL-18 activation (58). These include the release of alarmins such as HMGB1 and ASC *via* pyroptosis (13), FADD (Fas-Associated Death Domain) release *via* microvesicle shedding (59) or the degradation of UBE2L3 (E2 ubiquitin-conjugating enzyme L3) involved in NF-κB activation and pro-IL-1β turnover (27). We have shown that UBE2L3 degradation occurs in the absence of TLR-priming, as does FADD release (59), indicating the ability of active caspase-1 to cleave the same substrates independently of its priming state. This suggests that inflammasome activation can contribute to the amplification of the inflammatory response by mechanisms other than IL-18 signaling in an unprimed setting. This is not yet fully explored and needs to be further researched.

IL-18, unlike IL-1β, is constitutively expressed and is an important initiator of the inflammatory response through driving neutrophil recruitment, and further cytokine and chemokine release (60). IL-18 is involved in the progression of sterile inflammatory diseases including atherosclerosis, metabolic and neuro-inflammatory disorders (61). In these conditions, IL-18 mediates the activation of the NF-κB pathway and upregulation and secretion of TNF-α, adhesion molecules and chemokines (62). IL-18 is a key player in autoinflammatory syndromes, such as familial Mediterranean fever (FMF) caused by pyrin mutations (63) and the Macrophage Activation Syndrome (MAS) caused by NLRC4 gain of function mutations (64) that are characterized by systemically elevated IL-18 levels. IL-18 also plays a key role in the autoinflammatory Cryopyrin-associated periodic syndromes (CAPS) caused by mutations of NLRP3 (65), as IL-18R deletion lessens the disease phenotype to a greater extent than IL-1R deletion in a CAPS mouse model (66). Moreover, the release of damage associated signals such as ATP in transplantation can lead to P2X7R-mediated activation of the inflammasome, inducing the release of IL-18, but not IL-1β, which in turn plays an important role in allograft rejection through IFN-γ production and CD8^+^ T cell proliferation (67). This highlights the importance of IL-18 release by the rapid assembly of NLRP3 inflammasome in the initiation and progression of the inflammatory response.

Based on our results, we propose that the NLRP3 inflammasome in human monocytes is a “ready to assemble” complex, capable of propagating inflammatory responses at the early stages of inflammation when IL-1β has not yet been produced. The definition of priming in inflammasome research refers to transcriptional or post-translational changes required for NLRP3 to form an active inflammasome in response to a second signal. Based on our results it is possible that the second signal alone provides the licensing step required to allow NLRP3 oligomerization and consequent activation if NLRP3 is already present in the cell. However, we still know very little about what triggers this licensed state and the level of priming that cells present *in vivo*. Specifically what contributes to basal TAK1 and NF-κB activities is poorly characterized. Therefore, it is important to investigate the molecular mechanisms of NLRP3 activation in the absence of priming signals as this might reveal core regulatory mechanisms otherwise overshadowed by the different cellular processes and inflammatory status provided by diverse priming stimuli. We have shown the extent of NLRP3 activation varies between cell types; whether this is different between species, especially human and mouse has not been explored. One possible explanation to our results could be that humans are exposed to pathogenic and non-pathogenic danger signals throughout life and hence monocytes could exist in a basal primed state, allowing the inflammasome to assemble rapidly in response to danger. However, this remains poorly understood and requires further research in the future.

## Data Availability Statement

All datasets presented in this study are included in the article/**Supplementary Material**.

## Author Contributions

AG, SY, DB, and GL-C designed research. AG, SY, FM and ID-O, performed research. DD and EM-N contributed new reagents/analytic tools. AG, SY, FM-S, DB, and GL-C analyzed and discussed data. AG and GL-C wrote the paper. All authors contributed to the article and approved the submitted version.

## Funding

This work is supported by: a Medical Research Council PhD DTP studentship to AG (MR/N013751/1); a GSK funded PhD studentship to ID-O; a joint University of Manchester and China Council Scholarship (201608060031) and President’s Doctoral Scholar award to SY; a Medical Research Council grant (MR/N029992/1) to DB; a Wellcome Trust Investigator Award (110091) to DD and a Wellcome Trust and Royal Society Henry Dale Fellowship to GL-C (104192/Z/14/Z).

## Conflict of Interest

EN is an employee of the GSK group of companies.

The remaining authors declare that the research was conducted in the absence of any commercial or financial relationships that could be construed as a potential conflict of interest.
