# Corrigendum: Priming Is Dispensable for NLRP3 Inflammasome Activation in Human Monocytes *In Vitro*

**DOI:** 10.3389/fimmu.2021.763899

**Published:** 2021-09-13

**Authors:** Anna Gritsenko, Shi Yu, Fatima Martin-Sanchez, Ines Diaz-del-Olmo, Eva-Maria Nichols, Daniel M. Davis, David Brough, Gloria Lopez-Castejon

**Affiliations:** ^1^Division of Infection, Immunity and Respiratory Medicine, Faculty of Biology, Medicine and Health, Lydia Becker Institute of Immunology and Inflammation, University of Manchester, Manchester Academic Health Science Centre, Manchester, United Kingdom; ^2^Division of Neuroscience and Experimental Psychology, Faculty of Biology, Medicine and Health, Lydia Becker Institute of Immunology and Inflammation, University of Manchester, Manchester Academic Health Science Centre, Manchester, United Kingdom; ^3^Adaptive Immunity Research Unit, GSK, Stevenage, United Kingdom

**Keywords:** macrophage, inflammasome, NLRP3, priming, monocytes, IL-18, GSDMD

In the original article, there was an error in the section **Experimental Procedures**, **Cell Culture and Treatments**, Paragraph 2. The incorrect guide RNA oligonucleotide sequences were given for GSDMD knockout in THP-1 cells: “GSDMD knockout THP-1 cells were lentivirally generated using guide RNA oligonucleotides sequence 5′-CACCGCTGCAAGCTGGCCAGGTACC-3′ and 5′ AAACGGTACCTGGCCAGCTTGCAGC-3′ (22) by utilizing the lentiCRISPR v2 plasmid system. lentiCRISPR v2 was a gift from Feng Zhang (Addgene plasmid # 52961; http://n2t.net/addgene:52961; RRID : Addgene_52961)”.

A correction has been made to the above sentence, as follows: “GSDMD knockout THP-1 cells were lentivirally generated using guide RNA oligonucleotide sequences 5’-CACCGACCAGCCTGCAGAGCTCCAC-3’ and 5’-AAACGTGGAGCTCTGCAGGCTGGTC-3’ (22) by utilizing the lentiCRISPR v2 plasmid system. lentiCRISPR v2 was a gift from Feng Zhang (Addgene plasmid #52961; http://n2t.net/addgene:52961; RRID : Addgene_52961)”.

The authors apologize for this error and state that this does not change the scientific conclusions of the article in any way. The original article has been updated.

## Publisher’s Note

All claims expressed in this article are solely those of the authors and do not necessarily represent those of their affiliated organizations, or those of the publisher, the editors and the reviewers. Any product that may be evaluated in this article, or claim that may be made by its manufacturer, is not guaranteed or endorsed by the publisher.

