# Supplementary material for: Priming Is Dispensable for NLRP3 Inflammasome Activation in Human Monocytes In Vitro
Source: Front Immunol. 2020 Sep 30;11:565924. doi: 10.3389/fimmu.2020.565924 (PMC7555430; doi:10.3389/fimmu.2020.565924)
Supplement: Supplementary file 1 [file DataSheet_1.pdf]

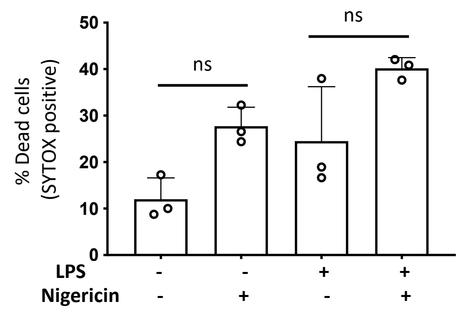

Figure S-1: THP-1 cell death following primed and unprimed inflammasome activation. THP-1 cells were left untreated or primed with LPS (1 µg/mL, 4h) prior to treatment with nigericin (10 µM, 45 min). Cells were then stained with SYTOX Green Stain to identify percentage of dead cells within the population. n=3 independent biological replicates, mean ± S.D. ns= not significant using one-way ANOVA comparing all groups.

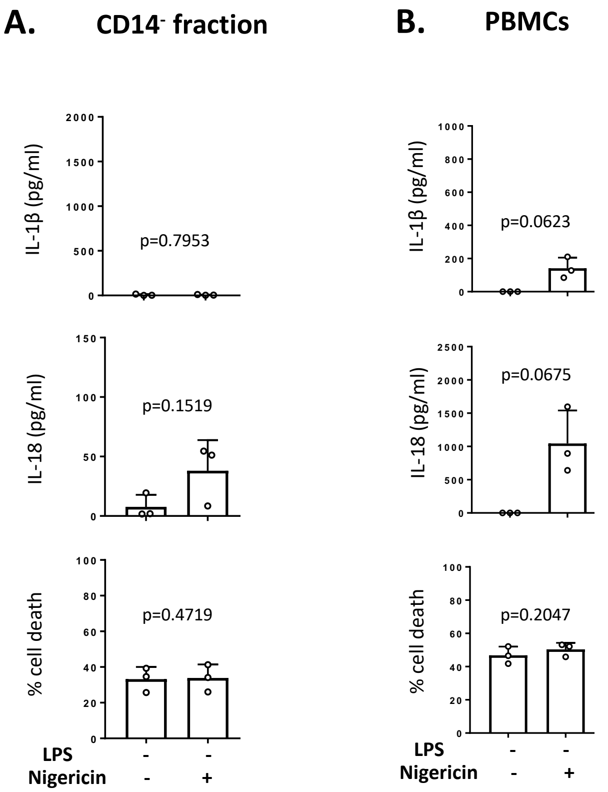

Figure S-2: CD14^{-} PBMC fraction as well as freshly isolated PBMCs show unprimed inflammasome activation. (A) The negative fraction from PBMC CD14 selection was collected and cultured with/without nigericin (10 μM, 45min) n=3 independent biological experiments (each from a different blood donor), mean ± S.D.. P values calculated using a paired t-test. (B) Freshly isolated PBMCs from healthy volunteers were cultured in the presence/absence of nigericin (10 μM, 45min). IL-1β and IL-18 were measured by ELISA and cell death was measured by LDH assay and shown as percentage relative to total cell death, n=3 independent biological experiments (each from a different blood donor), mean ± S.D.. P values calculated using a paired t-test.

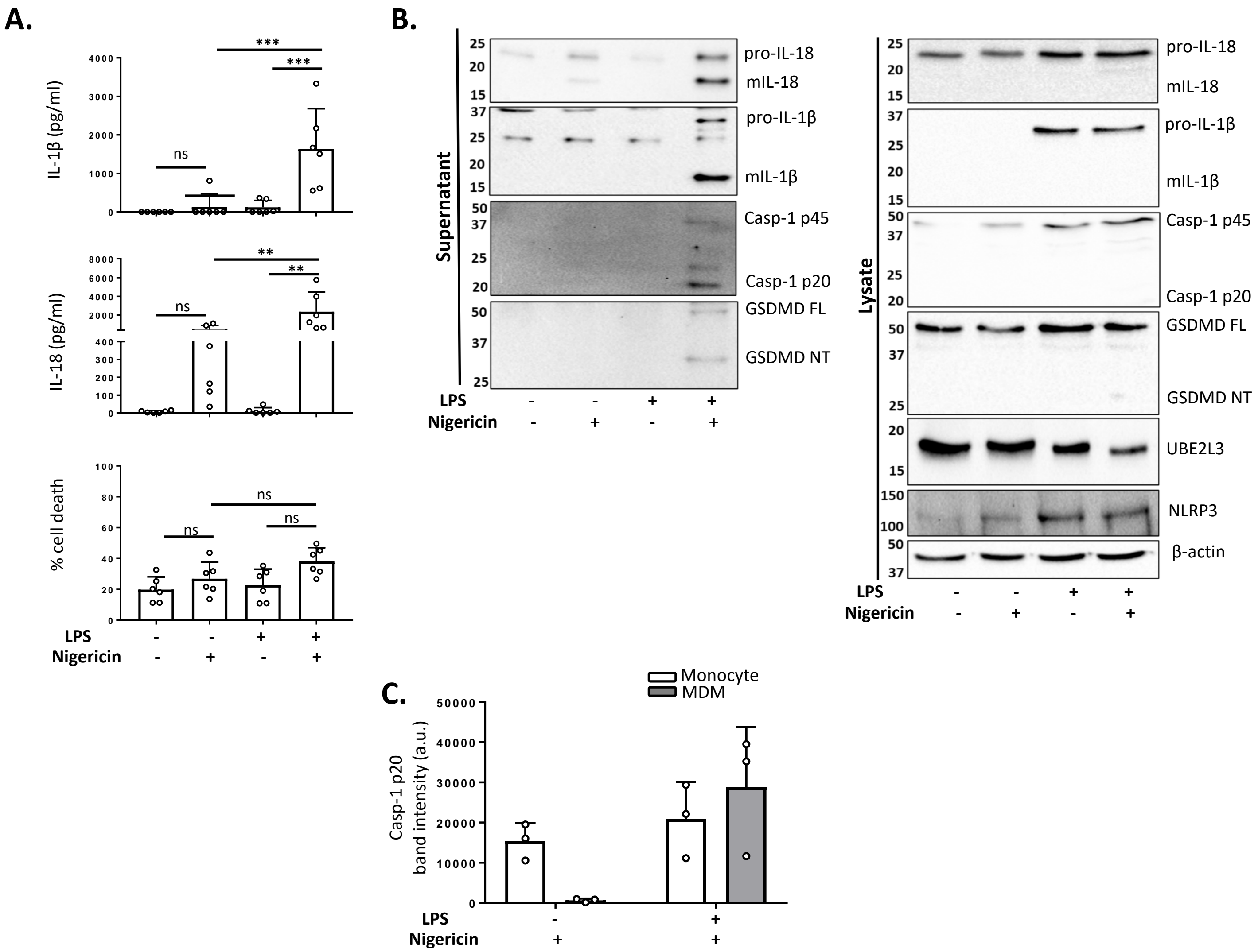

Figure S-3: Priming greatly potentiates NLRP3 inflammasome activation in human macrophages. MDMs were left untreated or primed with LPS (1 µg/mL, 4h) prior to treatment with nigericin (10 µM, 45min) to activate NLRP3 inflammasome. (A) IL-1β and IL-18 were measured by ELISA and cell death was measured by LDH assay and shown as percentage relative to total cell death, n=6 independent biological experiments (each from a different blood donor), mean ± S.D., ** = P < 0.01; *** = P <0.001 using one-way ANOVA comparing all groups. (B) Western blot analysis of MDMs for mIL-18 (18 kDa), pro-IL-18 (24 kDa), mIL-1β (17 kDa), pro-IL-1β (31 kDa), mCaspase-1 (20 kDa), pro-Caspase-1 (45 kDa), GSDMD full length (FL, 53 kDa), GSDMD N-terminus (NT, 31 kDa), UBE2L3 (17.9 kDa), NLRP3 (113 kDa), as well as loading control β-actin (42 kDa). Blots are representative of at least 3 independent biological experiments (each from a different donor). (C) The intensity of the released caspase-1 p20 bands by both primary human monocytes (data from Fig 1D) and MDMs was analysed using ImageJ (rsb.info.nih.gov).

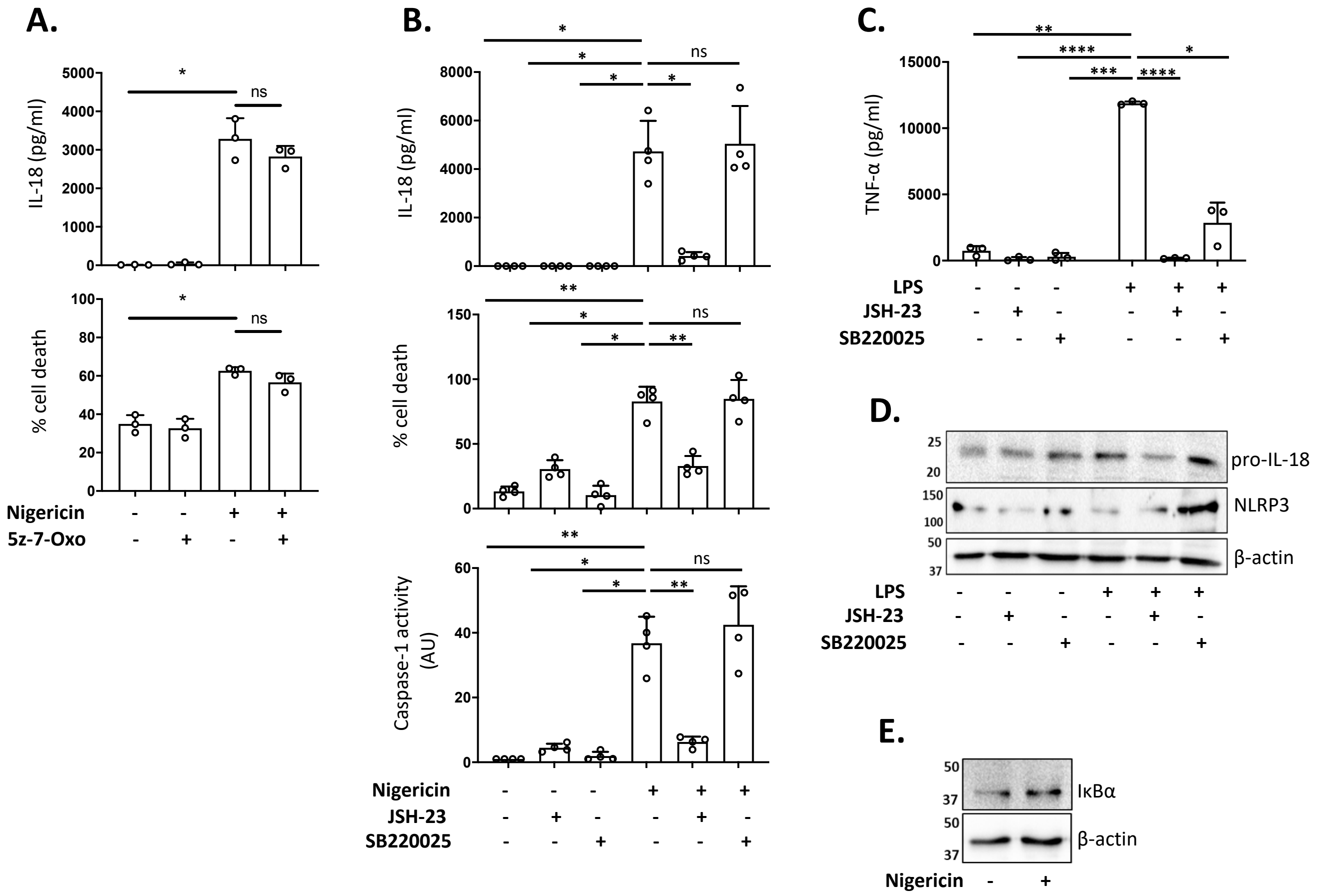

Figure S-4: TAK1 and NF-κB differentially contribute to unprimed inflammasome activation in THP-1 cells. (A, B) THP-1 cells were pre-incubated with 5z-7-Oxozeaenol (0.5 μM) for 15 minutes prior to treatment with nigericin (10 μM, 45 min). **(A)** Secreted IL-18 was measured by ELISA and cell death was measured by LDH assay and shown as percentage relative to total cell death. n=3 independent biological replicates. Error bars represent mean ± S.D., * = P < 0.05 using one-way ANOVA comparing each sample to nigericin only treated sample. **(B)** THP-1 cells were left untreated or pre-treated with JSH-23 (40 μM, 13h) or SB220025 (20 μM, 30 min) following treatment with nigericin (10 μM, 45min). Cell supernatants were assayed for the release of IL-18, cell death by LDH release and caspase-1 activity by caspase-1-Glo assay. n=4 independent experiments. * = P < 0.05; ** = P < 0.01 using one-way ANOVA comparing each sample to the nigericin only treated sample. (**C, D**) THP-1 cells were left untreated or pre-treated with JSH-23 (40 μM, 13h) or SB220025 (20 μM, 30 min) following treatment with LPS (1 μg/ml, 4h) or left untreated. **(C)** Cell supernatants were assayed for TNF-α release. n=3 independent biological replicates. Error bars represent mean ± S.D., * = P < 0.05; ** = P < 0.01; *** =P <0.001; **** =P <0.0001 using one-way ANOVA comparing each sample to the LPS only treated sample. **(D)** Lysates were analysed for pro-IL-18 (24 kDa), NLRP3 (113 kDa) as well as loading control β-actin (42 kDa). **(E)** Western blot analysis of THP-1 lysates with for IκBα (39 kDa) and loading control β-actin (42 kDa) from cells treated with nigericin (10 μM, 45min) or left untreated. All blots are representative of at least 2 independent biological experiments.

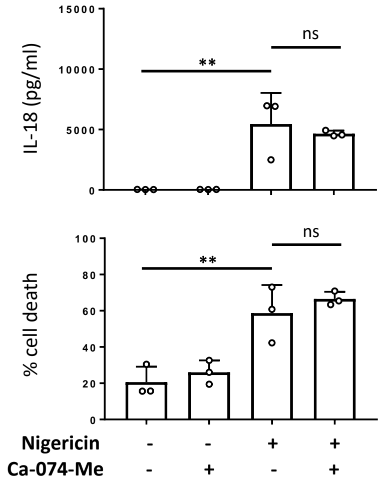

Figure S-5: Cathepsin-B inhibition with Ca-074-Me does not block the unprimed inflammasome response. Unprimed THP-1 cells were pre-incubated with Ca-074-Me (100 µM, 15 min) prior to treatment with nigericin (10 µM, 45 min). Secreted IL-18 was measured by ELISA and cell death was measured by LDH assay and shown as percentage relative to total cell death, n=3 independent biological replicates, mean ± SD, ** = $P < 0.01$; n.s. = non significant using one-way ANOVA comparing each sample to nigericin only treated sample.

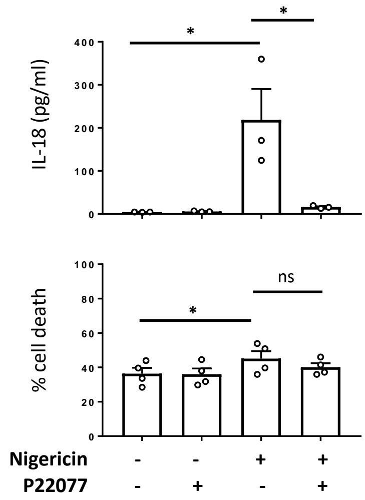

Figure S-6: Unprimed NLRP3 inflammasome activation in primary human monocytes is dependent on USP7/USP47 activity. Primary human monocytes were pre-incubated with P22077 (2.5 µM, 15 min) prior to treatment with nigericin (10 µM, 45 min). Secreted IL-18 was measured by ELISA and cell death was measured by LDH assay and shown as percentage relative to total cell death. n=3 independent biological replicates (each point represents a different blood donor), mean ± SD, * = $P < 0.05$; n.s.= non significant using one-way ANOVA comparing each sample to nigericin only treated sample.
